# Cerebellum-specific and age-dependent expression of an endogenous retrovirus with intact coding potential

**DOI:** 10.1186/1742-4690-8-82

**Published:** 2011-10-12

**Authors:** Kang-Hoon Lee, Makoto Horiuchi, Takayuki Itoh, David G Greenhalgh, Kiho Cho

**Affiliations:** 1 Department of Surgery, University of California, Davis, Sacramento, CA, USA; 2 Department of Neurology, University of California, Davis, Sacramento, CA, USA; 3 Shriners Hospitals for Children Northern California, Sacramento, CA 95817, USA

## Abstract

**Background:**

Endogenous retroviruses (ERVs), including murine leukemia virus (MuLV) type-ERVs (MuLV-ERVs), are presumed to occupy ~10% of the mouse genome. In this study, following the identification of a full-length MuLV-ERV by *in silico *survey of the C57BL/6J mouse genome, its distribution in different mouse strains and expression characteristics were investigated.

**Results:**

Application of a set of ERV mining protocols identified a MuLV-ERV locus with full coding potential on chromosome 8 (named ERV_mch8_). It appears that ERV_mch8 _shares the same genomic locus with a replication-incompetent MuLV-ERV, called Emv2; however, it was not confirmed due to a lack of relevant annotation and Emv2 sequence information. The ERV_mch8 _sequence was more prevalent in laboratory strains compared to wild-derived strains. Among 16 different tissues of ~12 week-old female C57BL/6J mice, brain homogenate was the only tissue with evident expression of ERV_mch8_. Further ERV_mch8 _expression analysis in six different brain compartments and four peripheral neuronal tissues of C57BL/6J mice revealed no significant expression except for the cerebellum in which the ERV_mch8 _locus' low methylation status was unique compared to the other brain compartments. The ERV_mch8 _locus was found to be surrounded by genes associated with neuronal development and/or inflammation. Interestingly, cerebellum-specific ERV_mch8 _expression was age-dependent with almost no expression at 2 weeks and a plateau at 6 weeks.

**Conclusions:**

The ecotropic ERV_mch8 _locus on the C57BL/6J mouse genome was relatively undermethylated in the cerebellum, and its expression was cerebellum-specific and age-dependent.

## Background

The concept of "endogenous" retroviruses (ERVs), which are inherited to subsequent generations by Mendelian order, was introduced following the discovery of three variants of ERVs in the genomes of laboratory mice and domestic fowls: murine leukemia virus (MuLV), mouse mammary tumor virus (MMTV), and avian leukosis virus [1,2]. ERVs are a family of long-terminal repeat (LTR) retrotransposons, and they occupy ~10% of the mouse genome [3,4]. In conjunction with the ERV population data accumulated from studies during the last few decades, the current mouse genome database renders an in-depth and systematic cataloguing of ERVs and other transposable and/or repetitive elements [4,5]. Mouse ERVs are segregated into three different classes (class I, II, III) based on the phylogenetic relatedness of their reverse transcriptase codons [6]. Class I (*e.g*., MuLV-type ERVs [MuLV-ERVs]), class II (*e.g*., MMTV-type ERVs), and class III ERVs represent ~0.7%, ~3%, and ~5.4% of the mouse genome, respectively.

Some studies have shed an initial light into the biological properties of mouse ERVs. Rowe *et al. *reported that activation of recombinant MuLV-ERVs is linked to the onset of thymic lymphomagenesis [7]. In addition, it has been demonstrated that extended culturing of embryonic cells derived from certain mouse strains, such as AKR mice, resulted in the *de novo *production and release of MuLV-type ERVs [8,9]. Recent studies have suggested that the envelope gene products of ERVs participate in various pathophysiologic processes, such as placental morphogenesis in mice and demyelination of oligodendrocytes in multiple sclerosis patients [10,11]. Our laboratory reported that stress signals elicited from injury and/or infection activate certain ERVs, and lipopolysaccharide treatment differentially induces the production and release of ERV virions from mouse primary lymphocytes of various origins and at different developmental stages [12-14]. Furthermore, it was observed that ERV expression patterns in mice are directly linked to ERV-, cell-, and/or tissue-type [14,15].

In this study, using a combination of different ERV mining protocols, a full-length MuLV-ERV locus with an intact coding potential was identified from the C57BL/6J mouse genome. The genomic distribution of this ERV in different mouse strains and its expression characteristics in various tissues, including different brain compartments, were investigated.

## Results

### Identification of a full-length MuLV-ERV locus on chromosome 8 of the C57BL/6J mouse genome

**Figure 1:**
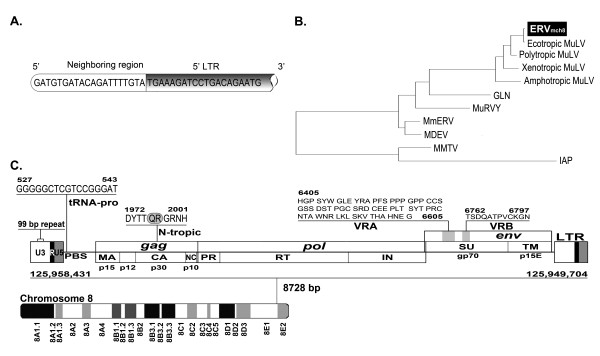
**Identification of a novel MuLV-ERV, named ERV_mch8_, on chromosome 8 of the C57BL/6J mouse genome**. **A**. The 40 nucleotide-probe, which was serendipitously identified during an ERV survey experiment, was used to mine new ERVs from the NCBI C57BL/6J mouse genome database. **B**. Phylogenetic relatedness of ERV_mch8 _with a diverse group of reference mouse ERVs is presented. ERV_mch8 _is highlighted (black box). Reference ERVs: ecotropic MuLV (U63133.1), polytropic MuLV (U13766), xenotropic MuLV (DQ399707), amphotropic MuLV (AF411814.1), GLN (AC136922), MuRVY (X87639.1), MmERV (AC005743), MDEV (AF053745), MMTV (AF228550.1), and IAP (AB099818.1). **C**. The genomic location of ERV_mch8 _mapped to chromosome 8 of the C57BL/6J genome. Functional features (proviral size, primer binding site, tropism motif, coding sequences [*gag*, *pro-pol*, and *env*], and LTR structure) of ERV_mch8 _are indicated on the proviral line drawing. The chromosome 8 ideogram was adopted and modified from the NCBI mouse genome database.

**Figure 2:**
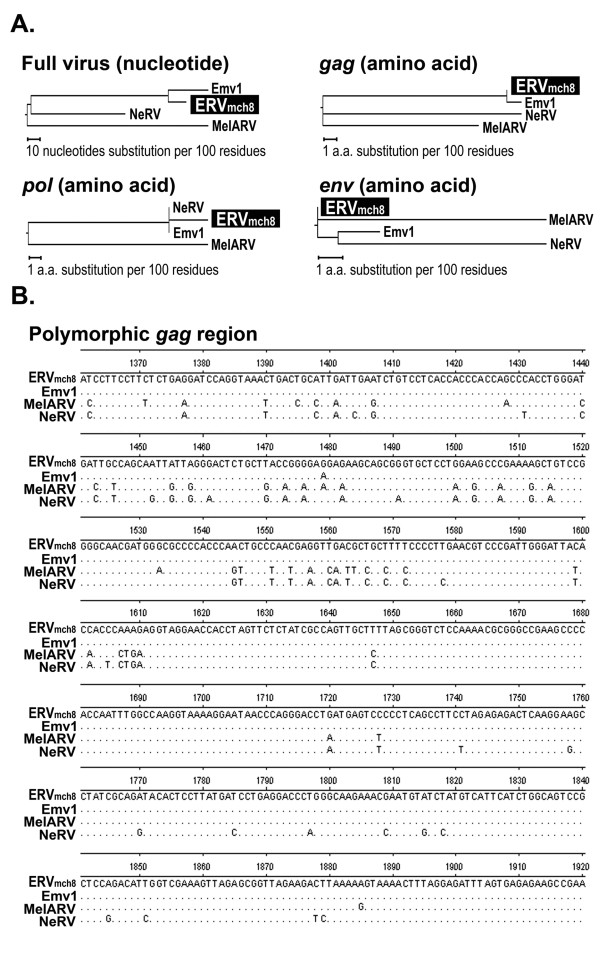
**Phylogenetic relatedness of ERV_mch8 _to three ecotropic mouse ERVs**. **A**. Phylogenetic relatedness of ERV_mch8 _with three mouse ecotropic ERVs, which have been characterized previously: complete nucleotide sequence (full provirus), *gag *polypeptide, *pol *polypeptide, and *env *polypeptide. MelARV (melanoma-associated retrovirus) (DQ366148.1), Emv1 (DQ366147.1), and NeRV (DQ366149.1) [17,18]. **B**. A main polymorphic region found in the *gag *p30 gene of ERV_mch8 _in comparison to the reference ecotropic ERVs is presented. Only the nucleotides different from the ERV_mch8 _sequence are indicated.

In our previous study, a stretch of 40 nucleotides at the junction of the envelope gene and 3' LTR of an unknown LTR retrotransposon was serendipitously identified during a genome-wide mining of MuLV-ERVs (Figure 1A) (unpublished). Using the 40 nucleotide sequence as an *in silico *probe, a combination of search programs, mainly NCBI BLASTN and BLASTP, was used to mine new ERV loci in the C57BL/6J mouse genome. Putative ERV loci identified from this mining experiment were subjected to an initial screening by an open reading frame (ORF) analysis and alignment against known ERVs. One putative full-length (8,728 nucleotides) MuLV-ERV was mapped on chromosome 8 (named "ERV_mch8_"), and it was determined to retain the intact coding potential for all three retroviral polypeptides (*gag *[537 amino acids], *pro-pol *[1,196 amino acids], and *env *[669 amino acids]) essential for virion assembly and replication (Figure 1B, C). In addition, there were two identical LTRs of 523 nucleotides, a tRNA^Proline ^primer binding site, and an N-tropic motif in p30 of the *gag *gene on the ERV_mch8 _locus [16]. Phylogenetic analyses using three reference MuLV-ERVs (Emv1, MelRV, and NeRV), which share high sequence similarities with ERV_mch8_, revealed that ERV_mch8 _retains one polymorphic cluster in the *gag *gene (Figure 2) [17-19]. According to previous reports, it appears that ERV_mch8 _shares the same genomic locus with another MuLV-ERV, called Emv2; however, this was not successfully confirmed because of an absence of relevant annotation and sequence information in the NCBI databases [20-22].

### Distribution of the ERV_mch8 _sequence in the genomes of laboratory and wild-derived mouse strains

**Figure 3:**
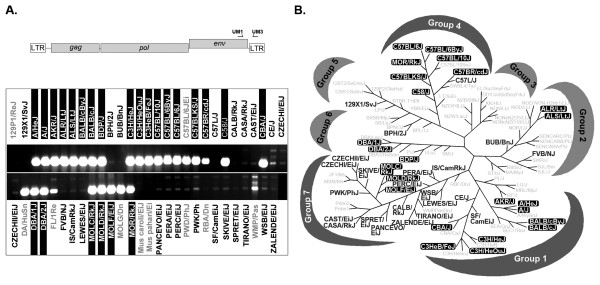
**Distribution of the ERV_mch8 _sequence in 57 different mouse strains**. **A**. A schematic drawing indicates the location of the primers used for PCR amplification of the ERV_mch8 _sequence. The distribution of the ERV_mch8 _locus in the genome of 57 mouse strains, both laboratory and wild-derived, was evaluated by PCR. **B**. The data regarding the distribution of the ERV_mch8 _sequence in various mouse strains are plotted onto a family tree developed by Petkov *et al. *[23]. Forty-seven of the 57 strains were mapped on the tree, which contains seven distinct groups, and each strain is highlighted with either a black box (evident presence of the ERV_mch8 _sequence) or in bold (no evident presence of the ERV_mch8 _sequence).

To determine the distribution of the ERV_mch8 _sequence in the genomes of laboratory and wild-derived mouse strains, genomic DNA samples isolated from 57 different strains were subjected to PCR genotyping using a primer set specific for the ERV_mch8 _sequence. The bands of the expected size were amplified in the vast majority of laboratory mouse strains, such as AKR/J and C3H/HeJ; conversely, they were present in only a limited number of wild-derived strains, such as MOLC/RkJ, MOLD/RkJ, and MOLF/EiJ (Figure 3A). The ERV_mch8 _sequence was not amplified in the pahari/Ei and caroli/EiJ strains, which are among the phylogenetically oldest wild-derived strains. Interestingly, the size and intensity of the bands, presumed to be amplified from the ERV_mch8 _sequences, were slightly variable depending on the mouse strain, suggesting polymorphisms in the sequences and/or copy numbers. Forty-seven of the 57 mouse strains were then mapped on Petkov *et al*.'s phylogenetic tree, which was established based on the profile of a set of single nucleotide polymorphism markers spanning the entire mouse genome, and is divided into seven distinct groups (Figure 3B) [23]. Interestingly, 16 of the 19 mouse strains mapped in Group 7 did not have evident amplification, whereas nine of the 11 in Group 1 as well as seven of eight in Group 4 had the expected bands (Figure 3).

### Brain-specific ERV_mch8 _expression

**Figure 4:**
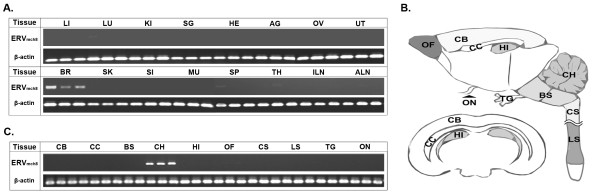
**Cerebellum-specific ERV_mch8 _expression in C57BL/6J mice**. **A**. Brain-specific expression of ERV_mch8 _was identified by surveying 16 different tissues (liver [LI], lung [LU], kidney [KI], salivary gland [SG], heart [HE], adrenal gland [AG], ovary [OV], uterus [UT], brain homogenate [BR], skin [SK], small intestine [SI], muscle [MU], spleen [SP], thymus [TH], inguinal lymph node [ILN], and axillary lymph node [ALN]). β-actin serves as a normalization control. **B**. The drawing illustrates the relative locations of the brain compartments and other neuronal tissues (cerebral cortex [CB], corpus callosum [CC], brain stem [BS], cerebellum [CH], hippocampus [HI], olfactory bulb [OF], cervical spinal cord [CS], lumbar spinal cord [LS], trigeminal ganglia [TG], and optic nerve [ON]) examined in this study. **C**. Cerebellum-specific expression pattern of ERV_mch8 _was identified by the comparison of 10 different neuronal tissues.

We then examined the expression pattern of ERV_mch8 _in a set of 16 selected tissues from female C57BL/6J mice (~12 weeks-old). No significant levels of expression were observed in any tissues examined except for the brain homogenates (Figure 4A). It needs to be noted that the brain homogenates were prepared using half of a brain from each animal. The findings from this experiment led us to speculate that the expression of the ERV_mch8 _might be specific for certain compartment(s) of the brain and other neuronal tissues. In addition to the six discrete compartments of the brain (cerebral cortex, corpus callosum, brain stem, cerebellum, hippocampus, and olfactory bulb), cervical and lumbar spinal cords, optic nerve, and trigeminal ganglia were separately collected from female C57BL/6J mice (~12 weeks-old) (Figure 4B) and were examined for the expression of ERV_mch8_. Interestingly, the evident expression of ERV_mch8 _was detected only in the cerebellum (Figure 4C). This cerebellum-specific pattern probably explains the variable expression levels of ERV_mch8 _in the brain homogenates processed from the half brains of three different mice, which may not represent the cerebellum proportionally (Figure 4A).

### Age-dependent regulation of the expression of ERV_mch8 _in the cerebellum

**Figure 5:**
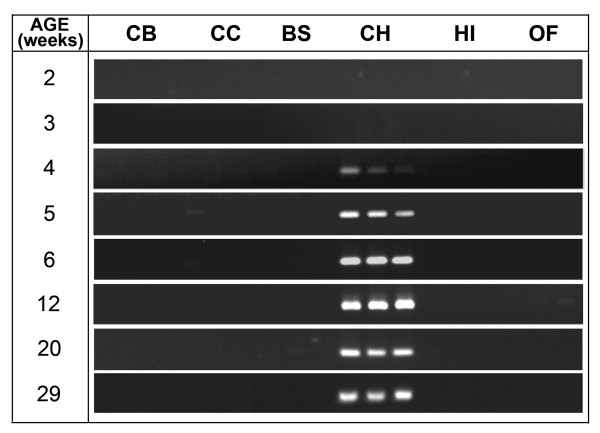
**Age-dependent ERV_mch8 _expression in the cerebellum**. Six different compartments of the brain (cerebral cortex [CB], corpus callosum [CC], brain stem [BS], cerebellum [CH], hippocampus [HI], and olfactory bulb [OF]) from eight different age groups of female C57BL/6J mice were surveyed for evidence of age-dependent/developmentally-regulated expression of ERV_mch8 _in the cerebellum.

In this study, we examined whether the cerebellum-specific expression of ERV_mch8 _is developmentally regulated using six different brain compartments (cerebral cortex, corpus callosum, brain stem, cerebellum, hippocampus, and olfactory bulb) from eight different age groups of female C57BL/6J mice, ranging from ~2 to ~29 weeks-old. No substantial expression of ERV_mch8 _was noted in the cerebellum until four weeks of age, and the expression plateaued at ~6 weeks of age (Figure 5). In contrast, there was no evident expression of ERV_mch8 _in the other brain compartments in all age groups examined. This finding suggests that the cerebellum-specific expression of ERV_mch8 _is age-dependent and potentially linked to the development of the cerebellum.

### Protein coding sequences neighboring the ERV_mch8 _locus

**Figure 6:**
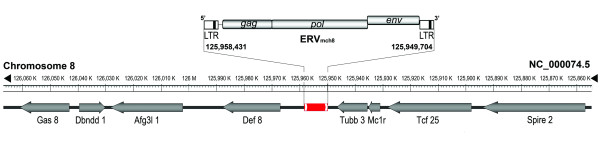
**The protein coding sequences neighboring the ERV_mch8 _locus on chromosome 8 of the C57BL/6J mouse genome**. A region of ~200 Kb on chromosome 8 of the C57BL/6J genome, which spans the area upstream and downstream of the ERV_mch8 _locus, was surveyed for protein coding sequences. The ERV_mch8 _locus, which resides on the minus strand, is marked in the center (red bar) and the minus strand is drawn on the top. Arrows indicate the transcriptional direction of the protein coding sequences. Please refer to the results section for the protein coding sequence abbreviations.

The transcription regulatory elements residing on the ERV sequences may participate in modulating the expression of neighboring protein coding sequences [24,25]. The genomic regions surrounding the ERV_mch8 _locus, 100 Kb upstream and 100 Kb downstream, were surveyed for annotated protein coding sequences on both strands. A total of eight protein coding sequences were identified: Spire2 (actin organizer), Tcf25 (transcription factor 25), Mc1r (melanocortin-1 receptor), Tubb3 (tubulin-β3), Def8 (differentially expressed in FDCP 8), Afg3l1 (ATPase family gene 3-like 1), Dbndd1 (dysbindin domain containing 1), and Gas8 (growth arrest specific 8) (Figure 6). Interestingly, the majority of these protein coding sequences were characterized to be associated with neuronal development and/or inflammation [26-31]. For example, Tubb3 and Spire2 are involved in processes responsible for brain development, while Mc1r plays a role in brain inflammation [32,33]. Further studies may confirm the possibility that ERV_mch8 _participates in the transcriptional control of some of these neighboring protein coding sequences.

### Unique methylation profile of the ERV_mch8 _locus in the cerebellum in comparison to the other brain compartments

**Figure 7:**
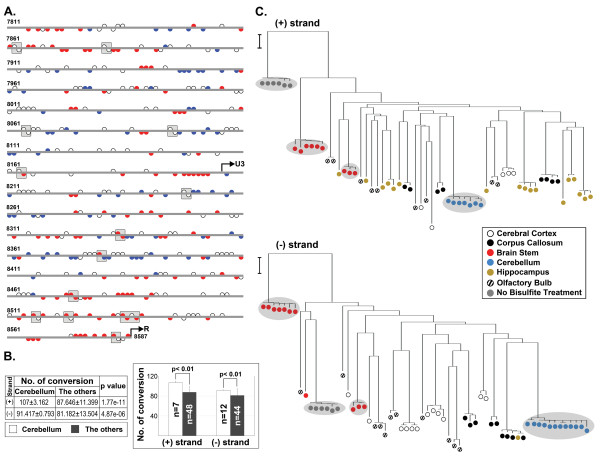
**Cerebellum-specific methylation pattern of the ERV_mch8 _locus**. **A**. The cytosine methylation profile of ERV_mch8 _in the cerebellum was compared to the methylation profile of an amalgamation of other brain compartments (cerebral cortex, corpus callosum, brain stem, hippocampus, and olfactory bulb). In particular, significant difference in the frequency of cytosine to thymine conversion (red half-circle) or in the frequency of no conversion (blue half-circle) in the cerebellum in comparison to the rest of the brain compartments is presented on both plus and minus strands. Open half-circle indicates no significant difference in the conversion frequency. Gray box identifies CpG dinucleotides. Start sites of U3 and R are indicated with an arrow. Plus strand cytosine residues of the ERV_mch8 _(minus strand of the chromosome orientation) are indicated by upward circles and minus strand with downward circles. **B**. The average number of converted cytosine residues in the ERV_mch8 _sequences isolated from the cerebellum was compared to the other brain compartments; values are summarized in a table and presented as a bar graph. N (number of sequences analyzed). **C**. Phylogenetic evaluation of the differentially converted ERV_mch8 _sequences was performed to compare the cytosine to thymine conversion profiles of the cerebellum and the other brain compartments. The bar on the left side of the tree represents the length of a nucleotide substitution per 100 residues.

In this study, we attempted to determine whether the cerebellum-specific expression of ERV_mch8 _is linked to the methylation status of its cytosine residues. The methylation profile within a segment of the ERV_mch8 _provirus in the cerebellum, spanning the 3'-end of *env *gene to the U3 sequence, was compared to a group of five other brain compartments (brain stem, cerebral cortex, corpus callosum, hippocampus, and olfactory bulb) from ~12 week-old C57BL/6J mice. At numerous nucleotide positions for both strands, a significantly higher frequency of cytosine to thymine conversion was observed in the cerebellum in comparison to the rest of the brain compartments (Figure 7A). The cerebellum also had a unique profile of no conversion of cytosines in comparison to the other brain compartments. In the cerebellum, the number of nucleotide positions with a significant conversion frequency (red half-circle) was substantially higher than the positions with a significant no conversion frequency (blue half-circle): plus strand (46 conversion positions vs. 23 no conversion positions) and minus strand (75 conversion positions vs. 63 no conversion positions). In addition, the average number of converted cytosine residues, thus unmethylated, in the ERV_mch8 _sequences isolated from the cerebellum was significantly higher (P < 0.01) compared to the rest of the brain compartments (Figure 7B). Furthermore, phylogenetic evaluation of the differentially converted ERV_mch8 _sequences was performed to compare the cytosine to thymine conversion profiles of the cerebellum and the other brain compartments (Figure 7C). Interestingly, the converted sequences isolated from the cerebellum formed a distinct branch for each strand: one branch had all seven plus strand sequences, and another branch contained all 12 minus strand sequences. Also, the converted sequences isolated from the brain stem were grouped into two small branches for each strand. The findings from these experiments suggest that the methylation profile of the ERV_mch8 _locus in the cerebellum is unique. Importantly, the number of unmethylated cytosine residues in the cerebellum was significantly higher compared to the rest of the brain compartments, which may be closely linked to the cerebellum-specific ERV_mch8 _expression.

## Discussion

Most characterized members of the C57BL/6J ERV population exist as multiple copies in the genome. A survey in this study identified only a single copy of an ecotropic ERV (ERV_mch8_) in the C57BL/6J mouse genome, and it is not currently annotated in the NCBI database (Build 37.1, as of November 12, 2010). ERV_mch8 _(8,728 nucleotides) shares a greater than 98% nucleotide sequence homology with the melanoma-associated retrovirus (MelARV), which was localized on chromosome 7 of the B16 melanoma cell line derived from the C57BL/6 mouse strain [18]. According to the results obtained from the *env *polypeptide alignment against MelARV, it appears that ERV_mch8 _harbors an ecotropic tropism trait. Pothlichet *et al. *reported that a single locus on chromosome 8-qE1 was mapped using the MelARV *env *sequence as a query and presumed that MelARV originated from the Emv2 locus, which is reported to be the only ecotropic ERV found in normal C57BL/6 cells [17,34]. Contrary to this report, the ERV_mch8 _locus, which has ~98% nucleotide sequence homology with MelARV, was mapped on the chromosome 8-qE1 junction, based on survey results using NCBI BLAST. In addition, Emv2 is located/annotated at 67.0 cM, ~11.4 cM upstream of the ERV_mch8 _locus (~78.4 cM), according to a survey of the NCBI map viewer [21,35]http://www.ncbi.nlm.nih.gov/projects/mapview. Thus, it is probable that ERV_mch8_, but not Emv2, is the probable progenitor of MelARV, if any. Unexpectedly, we were unable to retrieve the nucleotide sequence, which is presumed to be the Emv2 provirus, from the Emv2 locus annotated in the NCBI C57BL/6J mouse genome (Build 37.1, as of November 12, 2010) and MGI (MGI_4.4 as of November 19, 2010) databases. Further, we were unsuccessful in locating the Emv2 proviral sequence, either partial or full, using the keyword, "Emv2", in the NCBI Nucleotide database. However, it is still a possibility that ERV_mch8 _shares the same locus on chromosome 8-qE1 region with Emv2 with an assumption that the NCBI annotation information regarding the Emv2 locus needs to be revised.

Analysis of the distribution of the ERV_mch8 _sequence among various mouse strains demonstrated that a majority of strains in Groups 1 and 4 of the phylogenetic tree, which was developed by Petkov *et al*., harbor the proviral sequence in their genome. Within Group 1, which consists of mostly laboratory strains, including BALB/cJ and C3H, all except for the SF/CamEiJ and CE/J strains had evident amplification of the ERV_mch8 _sequence. The C57L/J strain in Group 4, which also contains the C57BL/6J strain, did not have the ERV_mch8 _sequence amplified, and this finding is consistent with the description from the Jackson Laboratory that "C57L/J mice carry no detectable endogenous ecotropic MuLV DNA sequences". On the contrary, there was no amplification of the ERV_mch8 _sequence in the vast majority (16 of 19) of Group 7, which is comprised of wild-derived strains. Interestingly, a unique branch of three strains (MOLC/RkJ, MOLD/RkJ, and MOLF/EiJ) in Group 7, which had the ERV_mch8 _sequence amplified, were derived by independent pairings of *Mus musculus molossinus *mice originating from Fukuoka, Japan (JAX^® ^NOTES Issue 456 and JAX Mice Database, Jackson Laboratory). The SPRET/EiJ mice, also from Group 7 and derived from wild mice caught in Puerto Real, Spain (JAX Mice Database, Jackson Laboratory), had no ERV_mch8 _sequence amplified. These findings suggest that the ERV_mch8 _sequence is present in wild mice originating only from certain geographic regions.

The unique methylation profile, in particular, the high number of converted cytosines in a segment of the ERV_mch8 _sequence of the cerebellum (~12 week-old mice) in comparison to the other brain compartments, may explain, at least in part, the cerebellum-specific expression of the ERV_mch8 _locus. Active transcription of this full-length MuLV-ERV (ERV_mch8_), presumed to retain the ecotropic tropism trait, from the age of five to six weeks may lead to a series of potential short-term and long-term events: 1) persistent expression of *gag*, *pol*, and *env *polypeptides, and their potential contribution to the biology of the cerebellum, 2) assembly of virus particles with ecotropic tropism followed by their release, and 3) very low-level, if any, infection (due to presumed to be poor replication-competency) of neighboring and/or distant cells expressing relevant receptor(s) during the course of the relatively long lifespan of brain cells [36].

## Conclusions

The key finding of this study that ERV_mch8 _expression is cerebellum-specific and age-dependent suggests that the expression of ERV_mch8 _is linked to the biology of the cerebellum. A set of further experiments is needed to unveil the detailed mechanisms controlling the cerebellum-specific and age-dependent expression of ERV_mch8_. In addition, a full investigation into the roles of ERV_mch8 _in the biology of the cerebellum and potentially other tissues is warranted.

## Methods

### Animals

Eight different age groups (~2 to ~29 weeks) of female C57BL/6J mice and ~12 week-old females were purchased from the Jackson Laboratory-West (West Sacramento, CA). The experimental protocol was approved by the Animal Use and Care Administrative Advisory Committee of the University of California, Davis. Three mice from each age group were sacrificed by CO_2 _inhalation or cervical dislocation followed by harvesting of different sets of tissues depending on the age groups. Certain brain samples were dissected further into their separate compartments and all tissue samples were snap-frozen.

### Genotyping PCR

Genomic DNA samples from 57 different inbred mouse strains (both laboratory and wild-derived) were purchased from the Jackson Laboratory (Bar Harbor, Maine). Genotyping PCR was performed using 100 ng of genomic DNA to determine the presence of ERV_mch8 _sequence using Taq polymerase from Qiagen (Valencia, CA) and a set of primers; UM1: 5'-GAA GTT GAA AAG TCC ATC ACT AA-3' and UM3: 5'-TCT GGG TCT CTT GAA ACT GT-3'.

### RNA isolation and RT-PCR

Total RNA was isolated from the tissue samples using an RNeasy Lipid Tissue Mini Kit (for brain tissues) or RNeasy Mini Kit (for non-brain tissues) from Qiagen. cDNAs were synthesized from 100 ng of total RNA using a QuantiTect Reverse Transcription Kit (Qiagen). A region near the 3'-end of the ERV_mch8 _transcript was amplified by PCR using the UM1 and UM3 primer set (see above). β-actin served as a normalization control. Primers for β-actin amplification are as follows; Forward: 5'-CCA ACT GGG ACG TGG AA-3' and Reverse: 5'-GTA GAT GGG CAC AGT GTG GG-3'.

### Genomic DNA isolation, bisulfite treatment, and PCR amplification

Genomic DNA was isolated from six different brain compartments (cerebral cortex, corpus callosum, brain stem, cerebellum, hippocampus, and olfactory bulb) of ~12 weeks-old mice using a DNeasy Tissue Kit (Qiagen). For the conversion of unmethylated cytosines to uracils/thymines, 2 μg of genomic DNA from each sample was treated with bisulfite using a Methyl Detector Kit (Active Motif, Carlsbad, CA). PCR was performed using Taq polymerase (Qiagen), 7.5 μl of bisulfite-treated DNA, and a set of primers; UM1 (see above) and m-L2D: 5'-CAA AAR RCT TTA TTR RAT ACA C-3'.

### Cloning and sequencing of PCR products

PCR products were purified using a QIAquick Gel Extraction Kit (Qiagen) followed by cloning into the pGEM^®^-T Easy vector (Promega, Madison, WI). Plasmid DNA was prepared using a QIAprep Spin Miniprep Kit (Qiagen) for sequencing at Functional Biosciences (Madison, WI).

### ERV mining, sequence alignment, and phylogenetic analyses

The National Center for Biotechnology Information (NCBI) BLASTN and BLASTP programs were alternately used for mining new ERVs from the C57BL/6J mouse genome database with a 40 nucleotide probe, which was serendipitously identified in our previous study (unpublished). Alignment and phylogenetic analyses of the DNA, including the bisulfite-converted DNA clones, and protein sequences were performed using the MegAlign program from DNASTAR (Madison, WI).

### Statistical analyses

The significance of differences in the C to T conversion rate at individual cytosine residue positions (plus and minus strands) was evaluated by Fisher's Exact probability test. The differences in the number of converted cytosine residues in the ERV_mch8 _sequence between the cerebellum and the other five brain compartments were evaluated by a Student's t-test. P values of less than 0.05 were determined to be significant.

## Competing interests

The authors declare that they have no competing interests.

## Authors' contributions

This study was conceived and managed by KC. DGG and TI participated in scientific discussions. KHL and MH performed the experiments and KHL generated the figures and drafted the manuscript. All authors read and approved the final manuscript.
